# P-195. Clinical characteristics, epidemiology and outcomes of imported malaria at a tertiary level hospital in Mexico

**DOI:** 10.1093/ofid/ofaf695.418

**Published:** 2026-01-11

**Authors:** Maximiliano Trevilla Viveros, Luis Gabriel Seriña Negrete, Maria Luisa Hernández Medel, Tiburcio Margarito Santos González, Joaquin Moreno Moreno, Jose Ivan Reyes Inclan, Silvia Paola Barragán Hernández, Sergio Bernardo Garcia Arce, Uzziel Aguilera Ontiveros, Cristian C Camilo Infante García

**Affiliations:** Hospital General de México "Dr. Eduardo Liceaga", Ciudad de México, Distrito Federal, Mexico; Hospital General de México "Dr. Eduardo Liceaga", Ciudad de México, Distrito Federal, Mexico; Hospital General de México "Dr. Eduardo Liceaga", Ciudad de México, Distrito Federal, Mexico; Hospital General de México "Dr. Eduardo Liceaga", Ciudad de México, Distrito Federal, Mexico; Hospital General de México "Dr. Eduardo Liceaga", Ciudad de México, Distrito Federal, Mexico; Hospital General de México "Dr. Eduardo Liceaga", Ciudad de México, Distrito Federal, Mexico; Hospital General de México "Dr. Eduardo Liceaga", Ciudad de México, Distrito Federal, Mexico; Hospital General de México "Dr. Eduardo Liceaga", Ciudad de México, Distrito Federal, Mexico; Hospital General de Mexico, Ciudad mexico, Distrito Federal, Mexico; Hospital General de Mexico, Ciudad mexico, Distrito Federal, Mexico

## Abstract

**Background:**

Mexico is a low malaria transmission setting, however, there has been an increase in the total number of imported cases, associated with greater migratory movement from Central and South America en route to the United States, most of which are linked to passage through the Darién Gap, a high-transmission area in the jungle region between Colombia and Panama.

**Figure:**
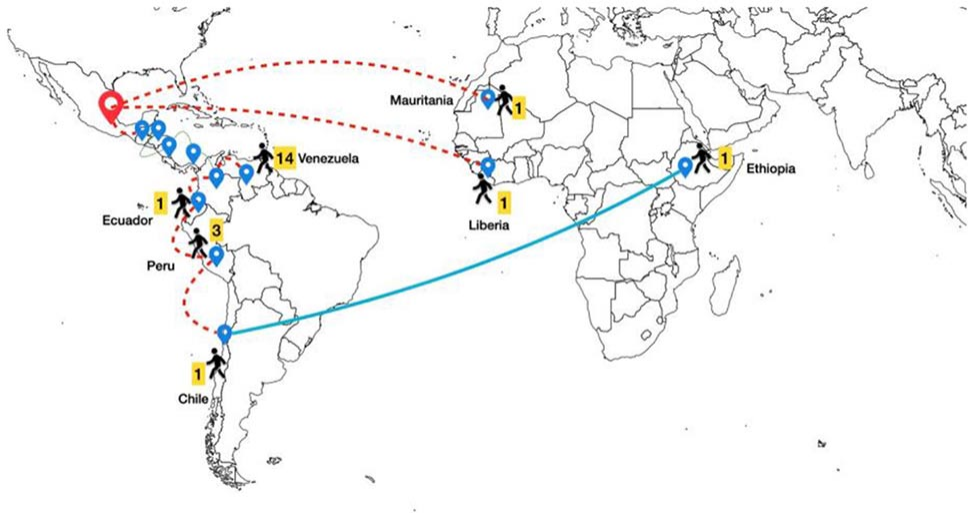
Migration Routes of Imported Malaria Cases, 2024

**Figure:**
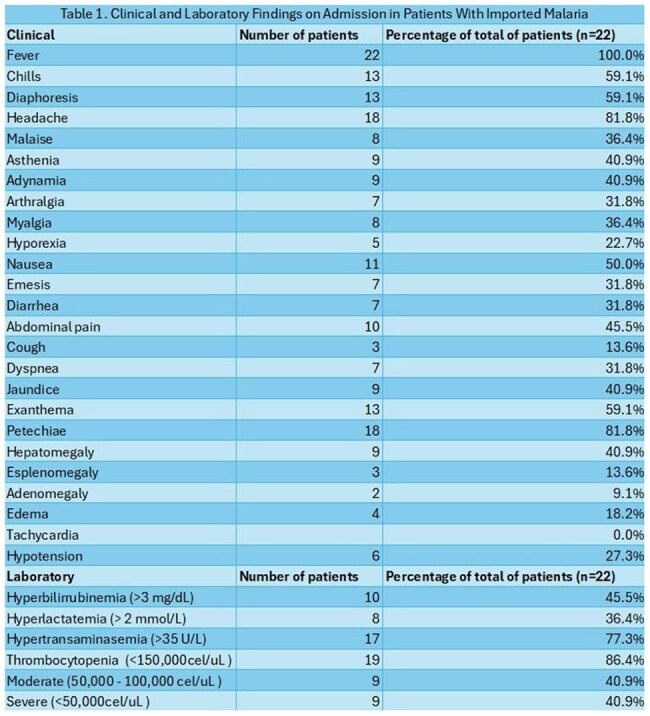

**Methods:**

Retrospective study of all imported malaria cases diagnosed from January 2024 to December 2024 in a tertiary-level hospital in Mexico City. All epidemiological, clinical and laboratory data were obtained from patients’ electronic medical records. All malaria cases were confirmed by thick blood smear examination in accordance with national guidelines. Severe malaria was defined according to the World Health Organization criteria.

**Figure:**
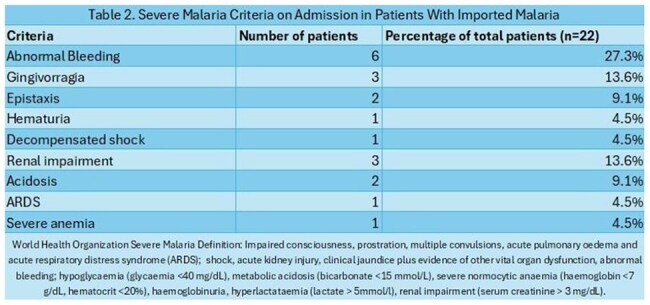

**Figure:**
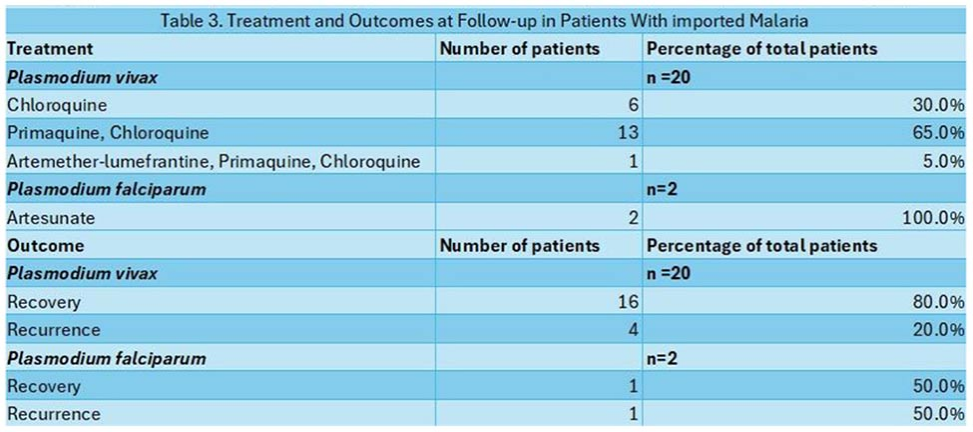

**Results:**

A total of 22 imported cases of malaria were admitted, most between May and August 2024 (54%). The patients were predominantly male (68%), with a mean age of 30.54 years. The most common route of arrival was through the Darién Gap (90%), with an average stay of 3.5 days in that region and a mean total travel time of 13.5 days. The most frequent country of origin was Venezuela (63%), followed by Peru (13%), and Chile, Ecuador and Ethiopia (4% each). There were 2 patients who flew directly from Mauritania and Liberia (Image 1). *Plasmodium falciparum* was found in both cases imported directly from Africa; the rest were identified as *Plasmodium vivax*. The most frequent clinical and laboratory findings were fever (100%) and thrombocytopenia (86%) respectively (Table 1). A total of 11 patients (50%) were classified as severe malaria, the most common complication being abnormal bleeding (Table 2). The average hospital stay was 7.5 days, and the main treatment was chloroquine in combination with primaquine for *Plasmodium vivax* (65%), and artesunate for *Plasmodium falciparum* (100%). The main outcome was recovery in both groups; however, recurrence was observed in patients who received inadequate treatment due to a shortage of antimalarial medication in the country (Table 3).

**Conclusion:**

Imported cases of malaria will continue to rise in Mexico with the current migration crisis; therefore, it is necessary to strengthen knowledge of this diagnosis and address the shortage of antimalarial medications.

**Disclosures:**

All Authors: No reported disclosures

